# Molecular Epidemiology and Variation of the BK Polyomavirus in the Population of Central and Eastern Europe Based on the Example of Poland

**DOI:** 10.3390/v14020209

**Published:** 2022-01-21

**Authors:** Jacek Furmaga, Marek Kowalczyk, Olga Furmaga, Christos A. Rokos, Tomasz Zapolski, Leszek Krakowski, Andrzej Jakubczak, Sławomir Rudzki

**Affiliations:** 1 Department of General and Transplant Surgery and Clinical Nutrition, Medical University of Lublin, 20-954 Lublin, Poland; jacekf61@o2.pl (J.F.); slawomirrudzki@umlub.pl (S.R.); 2 Institute of Quality Assessment and Processing of Animal Products, University of Life Sciences in Lublin, 20-950 Lublin, Poland; 3 Department of Radiology, General Hospital of Thessaloniki George Papanicolaou, 56403 Thessaloniki, Greece; ola.furmaga@gmail.com; 4 Department of Otolaryngology, Head and Neck Surgery, AHEPA Hospital, Aristotle University of Thessaloniki, Kiriakidi 1, 54636 Thessaloniki, Greece; christos.rokos@gmail.com; 5 Department of Cardiology, Medical University of Lublin, 20-954 Lublin, Poland; zapolia@wp.pl; 6 Department and Clinic of Animal Reproduction, Faculty of Veterinary Medicine, University of Life Sciences, Gleboka 30, 20-612 Lublin, Poland; leszek.krakowski@up.lublin.pl; 7 Institute of Biological Basis of Animal Production, Faculty of Animal Sciences and Bioeconomy, University of Life Sciences in Lublin, 20-950 Lublin, Poland

**Keywords:** BKPyV, molecular diagnostics, polymorphism, genotyping

## Abstract

The BK polyomavirus (BKPyV) is a widespread pathogen in humans. Polymorphism of the region encoding the VP1 protein of BKPyV provides the basis for classifying the virus into types and subtypes, whose frequency varies depending on geographic location. The aim of our study was to determine the frequency of BKPyV in the Polish population and to assess its variation by analysing polymorphism in the typing region. The study was conducted on 168 healthy, Polish volunteers, whose blood (plasma) and urine were sampled. The virus was detected using PCR, products, sequenced and subjected to bioinformatic analysis. In addition, viral load was assessed by qPCR. The presence of the genetic material of the BK virus was noted in 61/168 urine samples but in none of the plasma sample. Sequencing and phylogenetic analysis confirmed that the BKPyV isolates were of types I and IV, dominant in Europe (63.93% and 36.07%, respectively). All isolates from genotype I belonged to subtype Ib-2, showing polymorphism at position 1809 with a frequency of 61.54% (G1809A) and 38.46% (G1809C). To the best of our knowledge, this is the first study of this magnitude on the genetic variation of BKPyV among healthy volunteers in Poland.

## 1. Introduction

The BK polyomavirus (BKPyV) was first isolated and described in the 1971 by Gardner et al. [1]. Its name is derived from the initials of the patient in which it was first diagnosed. Antibodies against BKPyV are currently detected in more than 80% of the population tested [2,3]. High frequency of the virus is also confirmed by the presence of its genetic material in the urine of over 12% of healthy subjects [4].

Infection with the pathogen usually takes place during childhood [5]. Following primary infection, the virus replicates in the respiratory tract. It can infect the upper respiratory tract and cause fever, but infection is usually asymptomatic [6,7], and after reaching the target sites in the renal tubules or the epithelium of the bladder, it enters a state of latency. The course of infection can be severe in individuals with impaired immunity, especially in kidney transplant or haematopoietic stem cell transplant recipients. Due to impairment of immune processes caused by immunosuppressive drugs, the virus actively replicates in the epithelial cells, and ‘decoy cells’ are released into the urine. Reactivation of infection is initially asymptomatic, with asymptomatic viruria followed by viraemia when viral particles enter the bloodstream [8]. In such patients, reactivation of the virus or its acquisition via transplant can cause a number of serious complications, including BK polyomavirus-associated nephropathy (BKPyVAN), leading to loss of the graft following a kidney transplant or severe haemorrhagic cystitis (HC) following an allogeneic haematopoietic stem-cell transplant (HSCT) [5,9,10,11,12,13]. For this reason, the presence and rate of replication of BKPyV should be monitored in recipients. Methods used for this purpose include polymerase chain reaction (PCR), enabling detection of the genetic material of the virus in the urine and blood of patients [11,14].

**Figure 1:**
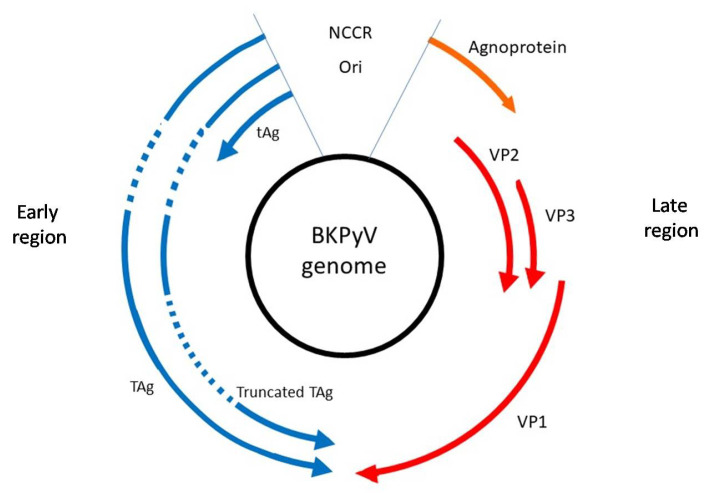
Genomic organization of BK Polymomavirus (BKPyV). The late coding regions encode structural proteinsVP1, VP2, VP3 and the nonimmunogenic agnoprotein. The early coding regions encode the large tumour antigen (TAg), small tumour antigen (tAg) and truncated T antigen (truncTAg).

BKPyV is a non-enveloped dsDNA virus with a 5100 bp genome encapsulated in icosahedral virions containing an early region, the late region and non-coding control region—NCCR (Figure 1). Control region contains the site of initiation of replication and regulates bidirectional expression of the coding regions and the viral DNA replication. Transcription extends directionally from initiation sites near the origin to produce the early and late messenger RNAs, which are transcribed from the opposite strands of the viral genome [15]. The early region includes the genes coding for the large tumour antigen (TAg), small tumour antigen (tAg) and truncated T antigen (truncTAg). Both TAg, and tAg are products of two alternatively spliced mRNAs and are the first proteins expressed during infection [16]. Moreover, Abend et al. [17] confirmed the expression of protein (17–20 kDa) from an alternatively spliced mRNA of the early region, a truncated form of large tumour antigen, which may play a role in the cell transformation [18].

The late region encoding structural proteins VP1, VP2 and VP3 building the capsid, and a small regulatory protein–agnoprotein, which is believed to contribute to the overall viral replication cycle [18]. Genes of late region are expressed after the onset of viral DNA replication [17]. The major capsid protein VP1 forms the outer shell of the virions and take part in the attachment of the virus to the cell surface receptors, facilitating the virus’s spread between the cells. VP1 is responsible for the antigenic properties of the virus. It can be divided into five outer loops, BC, DE, EF, GH and HI. The BC loop contains the region with nucleotides at positions 1744–1812, used to identify the four main BKPyV genotypes on the basis of nucleotide polymorphisms [19,20]. VP1 has an essential role in host-cell receptor binding and changes in the BC loop. It can change the biological properties of the virus and increase its pathogenicity [21,22]. VP2 and VP3 have overlapping sequences and are located at the inner parts of the capsid. These proteins play important role in the nucleation of the viral DNA into the capsids during the encapsidation process [18].

Analyses of the molecular epidemiology of BKPyV indicate that the most common type globally occurring is type I, which includes subtypes Ia (detected mainly in African populations), Ib-1 (detected in southeast Asia), Ib-2 (dominant in Europe and western Asia) and Ic (northeast Asia) [23,24,25]. The second most common type is type IV, diagnosed mainly in Europe and Asia [25,26], while genotypes II and III are detected much less often. Geographic variation in genotypes of the BKV virus indicates that, as in the case of the JC virus (another representative of Polyomaviridae), it can be used as a marker in molecular anthropology and study of the directions of human migration [27,28,29]. Tracing of migration may be facilitated by the data concerning rate of substitutions, which within BKPyV ranged from 9.68 × 10^−8^ to 1.93 × 10^−7^ substitutions per site per year (data for entire coding region) [23]. Analysis of polymorphism between isolates is also important in medical research because molecular variation in BKPyV may entail changes in tropism and also influence the clinical manifestation of infection. Studies show that high variation in the BC loop of the VP1 protein can lead to the emergence of quasi-species that may be associated with higher pathogenicity [30] and affect parameters such as infectivity and resistance to neutralization by antibodies [31].

Knowledge of the molecular epidemiology of the BKPyV is unquestionably useful in medical science (association between genetic variants of the virus and the clinical situation of the patient) as well as evolutionary research (directions of evolution and selection acting on the pathogen). Holistic analysis of circulation of BKPyV requires more complete global maps of infections and identification of variants occurring in different parts of the world. There are few reports on the genetic structure of BKPyV in the countries of Central and Eastern Europe, and therefore it is worthwhile to add data from these areas to the global map of infections with BKPyV. The aim of the study was to identify and assess polymorphism of BKPyV and the viral load in healthy volunteers from the Polish population and to compare the results with the resources of global bioinformatics databases.

## 2. Materials and Methods

### 2.1. Study Population

From December 2017 to August 2018, at the Department of General and Transplant Surgery and Clinical Nutrition, Medical University of Lublin (Poland), 200 individuals of both sexes were initially selected for the study. Individuals without symptoms of immunodeficiency were included, and those with acute febrile disease, urinary tract infections, hepatitis B or C, HIV, or LE were excluded, as well as patients treated with immunosuppressants. Ultimately, after informed written consent had been obtained from all subjects, a total of 168 urine and plasma samples were collected during routine examinations from 110 men aged 21 to 75 years (average age 49) and from 58 women aged 27 to 75 (average age 55). The samples were stored at −80 °C until analysis. Ethical permission for the research was granted by the Bioethics Committee of the Medical University of Lublin, Poland (KE-0254/281/2017).

### 2.2. DNA Extraction

DNA was extracted from the blood and urine with the DNeasy Blood & Tissue Kit (Qiagen Hilden, Germany). In the case of blood, DNA was extracted from 200 µL of blood. Some modifications were used to extract DNA from urine. Urine samples were centrifuged at 14,000× *g* for 10 min, and DNA was extracted from the resulting pellets suspended in 200 µL of free urine. Samples were subjected to lysis in 200 µL AL buffer and 20 µL proteinase K at 56 °C with shaking for 10 min. Further isolation steps were performed according to the manufacturer’s protocol. DNA was eluted with 100 µL of AE elution buffer.

### 2.3. PCR Amplification

After extraction, each DNA sample was verified by amplification with primers specific to human β-actin [32] to avoid false negative results, which can result from ineffective amplification or the presence of PCR reaction inhibitors.

PCR was used to detect BKPyV genetic material in the samples. The primers used amplified a partial sequence of the VP1 gene, spanning from 1630 bp to 1956 bp in sequence NC_001538 from the NCBI database [28]. The targeted region contains the typing region used for genotyping of BKPyV variants.

The primer sequences, reaction conditions and mixture composition are given in Table 1 and Table 2. The optimum amplification temperature was established by modifying the reaction conditions using a thermal gradient (50–60 °C) and a magnesium concentration gradient (1.5–3 mM). The optimum amplification temperature was determined to be 54 °C for primers targeting the genetic material of BKPyV and 56 °C for primers targeting β-globin. The optimum magnesium concentration for both reactions was established at 2.5 mM, tested samples were subjected to 40 cycles of PCR. PCR products were separated in a 2% agarose gel with ethidium bromide at 70 V. The lengths of the bands were determined by comparison to GeneRuler 100 bp size markers (Thermo Fisher Scientific, Foster City, CA, USA). The electrophoresis results were analysed under UV light with Scion Image software (Scion Corporation, Frederick, MD, USA).

### 2.4. Sequencing and Bioinformatic Processing

Amplification products were purified using the ExoSAP-IT kit (Affymetrix, Santa Clara, CA, USA). Sequencing PCR was performed with the same primers as for the original amplification, using the BigDye^®^ Terminator 3.1 CycleSequencing Kit (Thermo Fisher Scientific, Foster City, CA, USA) as recommended by the manufacturer. Sequencing PCR products were purified with the ExTerminatorKit (A&A Biotechnology, Gdynia, Poland). Samples were subjected to thermal denaturation in formamide. The sequencing reaction was carried out in a 3100 Avant Genetic Analyser (Applied Biosystems, Foster City, CA, USA). To avoid sequencing errors, both sequenced strands were analysed.

Sequencing results were analysed using DNA Baser software. Editing, alignment of sequences, localisation of polymorphisms and assignment to types were performed using MEGA 11 and Bioedit software. Phylogenetic analysis was carried out using MEGA11 by the neighbour-joining (NJ) algorithm with Kimura’s two-parameter distance method and a bootstrap value of 1000.

BKPyV genotypes were determined by comparing the sequences obtained to sequences from the NCBI database. BKPyV isolates were classified into genotypes on the basis of polymorphic nucleotides proposed by Randhawa et al. [34], using an algorithm designed by Morel et al. [35] and phylogenetic analysis based on nucleotide sequences. Sequences obtained during this study are available in GenBank with the accession numbers: OM179842 (Ib-2_POL_K), OM179843 (Ib-2_POL_F), OM179844 (POL-IV)

### 2.5. Quantitation of BK Virus DNA by Real-Time Polymerase Chain Reaction

Quantitative assessment of BKPyV was conducted by qPCR, using the GeneProof BK/JC Virus PCR Kit in an ABI Prism^®^ 7500 Real-Time PCR System (Applied Biosystems), according to the kit manufacturer’s instructions. BKPyV detection is based on amplification of a specific conservative DNA sequence overlapping the boundary between the genes for the VP1 and VP2 proteins. The presence of viral genetic material is indicated by the increase in FAM fluorophore fluorescence. The reaction was prepared in a 40 µL volume (30 µL of Reaction Mix and 10 µL of DNA). The qPCR reaction conditions were as follows: UDG incubation at 37 °C for 2 min, DNA hot start polymerase activation at 95 °C for 10 min, followed by 45 cycles of DNA melting at 95 °C for 5 s, annealing at 60 °C for 40 s, and extension at 72 °C for 20 s. Serial dilutions of 2 × 10^4^ copies/μL, 2 × 10^3^ copies/μL, 2 × 10^2^ copies/μL, and 2 × 10^1^ copies/μLwere used to prepare the standard curve, which was used to determine the number of copies of the virus based on the Ct value. Negative control reactions without DNA and a positive control supplied by the manufacturer were also included in every run. The samples were run in duplicate. Viral load was expressed as the number of BKPyV copies per ml of urine. After extraction, each DNA sample was verified by amplification with primers specific to human β-actin [32] to avoid false negative results, which can result from ineffective amplification or the presence of PCR reaction inhibitors.

### 2.6. Statistical Analysis

The results of quantitative analysis of viral load were analysed by analysis of variance (ANOVA), to determine whether there were any statistically significant differences between the means. The calculations were made using the SAS statistics package (SAS Institute, Cary, NC, USA). Statistical significance was established as *p* ≤ 0.05.

## 3. Results

**Figure 2:**
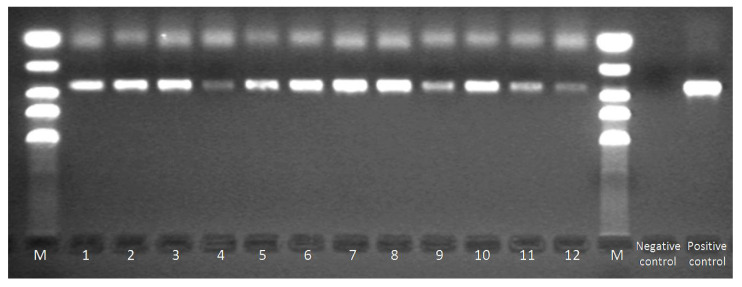
Electrophoretic separation products of PCR with primers targeting typing region of BKPyV: M-marker (bands 50 bp, 200 bp, 400 bp, 600 bp, 1000 bp), 1–12-BKPyV positive samples (bands of 327 bp confirms the presence of BKPyV genetic material), negative and positive control.

The study included 168 healthy individuals aged 21 to 75 (average age 51.2). The presence of the genetic material of BKPyV in the urine was confirmed in 61/168 of cases (36.31%), including 22/58 in women and 39/110 in men (37.93% and 35.45%, respectively) (Figure 2, Table 3).

The average age of the subjects with confirmed infection was 53.2 and did not differ significantly between women and men (*p* = NS). BKPyV genetic material was not found in any of the plasma samples.

PCR with primers flanking the β-globin gene confirmed the effectiveness of DNA isolation in each of the test samples as well as the absence of PCR inhibitors.

The isolates were classified into genotypes based on SNPs in the VP1 coding sequence (Table 4). The results indicated that the isolates belonged to genotypes I and IV, while the presence of genotypes II and III was not noted (39/61, 22/61, 0/61, and 0/61; 63.93%, 36.07%, 0%, and 0%, respectively).

**Figure 3:**
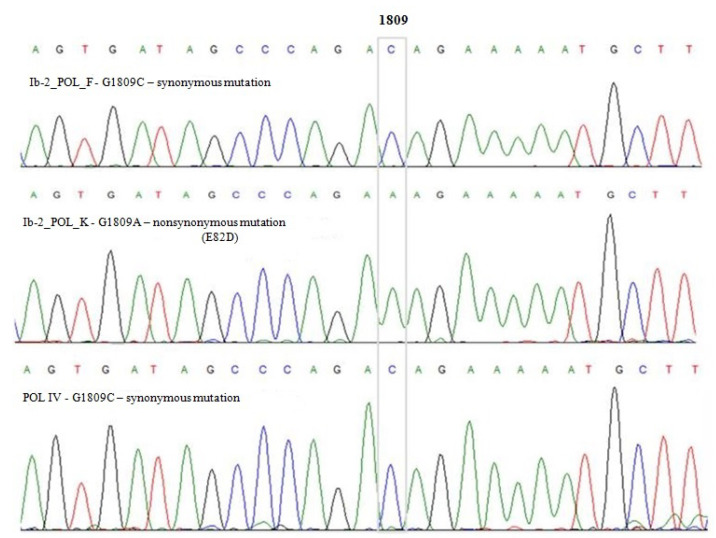
Sequencing results showing polymorphism G1809A, which was the basis for distinguishing variants Ib-2_POL_K and Ib-2_POL_F.

In the sequence analysed there were 22 nucleotides distinguishing types I and IV (G1704A, C1716T, C1722T, G1744A, A1746T, A1747G, T1760A, A1769G, G1770A, G1775C, A1784C, A1787C, A1792G, G1793A, C1848A, C1851A, C1854T, A1860G, C1869T, G1890A, A1905G, and C1912A–10 synonymous substitutions and 12 nonsynonymous substitutions), five polymorphisms distinguishing subtype Ia from Ib (G1687C, T1698A, G1809A/C, T1908A, and T1923C) and two polymorphisms distinguishing subtype Ib-1 from Ib-2 (G1687C and T1908A). The location of the polymorphisms is consistent with results previously reported by Ikegaya et al. [28]. Analysis of the sequences belonging to genotype I revealed a substitution of adenine or cytosine for guanine at position 1809 (G1809A/C), which provided the basis for distinguishing two variants, designated Ib-2_POL_K (G1809A) and Ib-2_POL_F (G1809C) (Figure 3). The G1809A substitution was nonsynonymous and led to a change in amino acids in the protein sequence (glutamic acid to aspartic acid).

The highest number of positive results was obtained in subjects between the ages of 41 and 70, while younger subjects aged 21–40 accounted for less than 20% of all those infected (Table 5). The highest viral load was detected in subjects between the ages of 21 and 30; this was two orders of magnitude higher than in the 41–60 age group and one order of magnitude higher than in the 61–70 age range (Table S1). Quantitative analysis of BKPyV viruria showed that it ranged from 1.2 × 10^2^ to 9.1 × 10^6^, with an average of 2.16 × 10^5^, and was lower in women than in men (1.42 × 10^5^ and 2.58 × 10^5^, respectively, *p* = NS—Table S2). Differences were observed in the number of copies detected in the case of different BKPyV variants, with a statistically significant difference in viral load between genotypes I and IV (3.24 × 10^5^ and 2.40 × 10^4^, respectively, *p* < 0.05). Within subtype Ib-2 in our group of subjects, Ib-2_POL_K and Ib-2_POL_F did not differ significantly in terms of the number of copies detected (3.14 × 10^4^ and 7.93 × 10^5^, respectively, *p* = NS).

The most commonly detected genotype in our population was Ib-2, found in 63.93% of the entire study group, with a frequency of 54.55% in women and 69.23% in men, and clear dominance over genotype IV, found in 36.07%, 45.45% and 30.77%, respectively. The dominant variant within genotype I was Ib-2_POL_K, accounting for 61.54%. The very high percentage of variant Ib-2_POL_F, both in the entire group and separately in women and men (38.46%, 33.33% and 40.74%, respectively) strongly suggested that group Ib-2 could potentially be divided into two variants depending on the G1809A/C mutation, and this division was made.

After genotype I, the most commonly identified BKPyV genotype in our subjects was genotype IV, which was also present in a very high percentage of cases in the entire group as well as in women and men separately: 36.07%, 45.45% and 30.77%. An increase in the number of cases detected with increasing age, in agreement with the global tendency, was evident in the case of Ib-2_POL_K. No such tendency was observed in the isolates with genotype IV or variant Ib-2_POL_F, whose frequency remained constant and did not increase with age.

**Figure 4:**
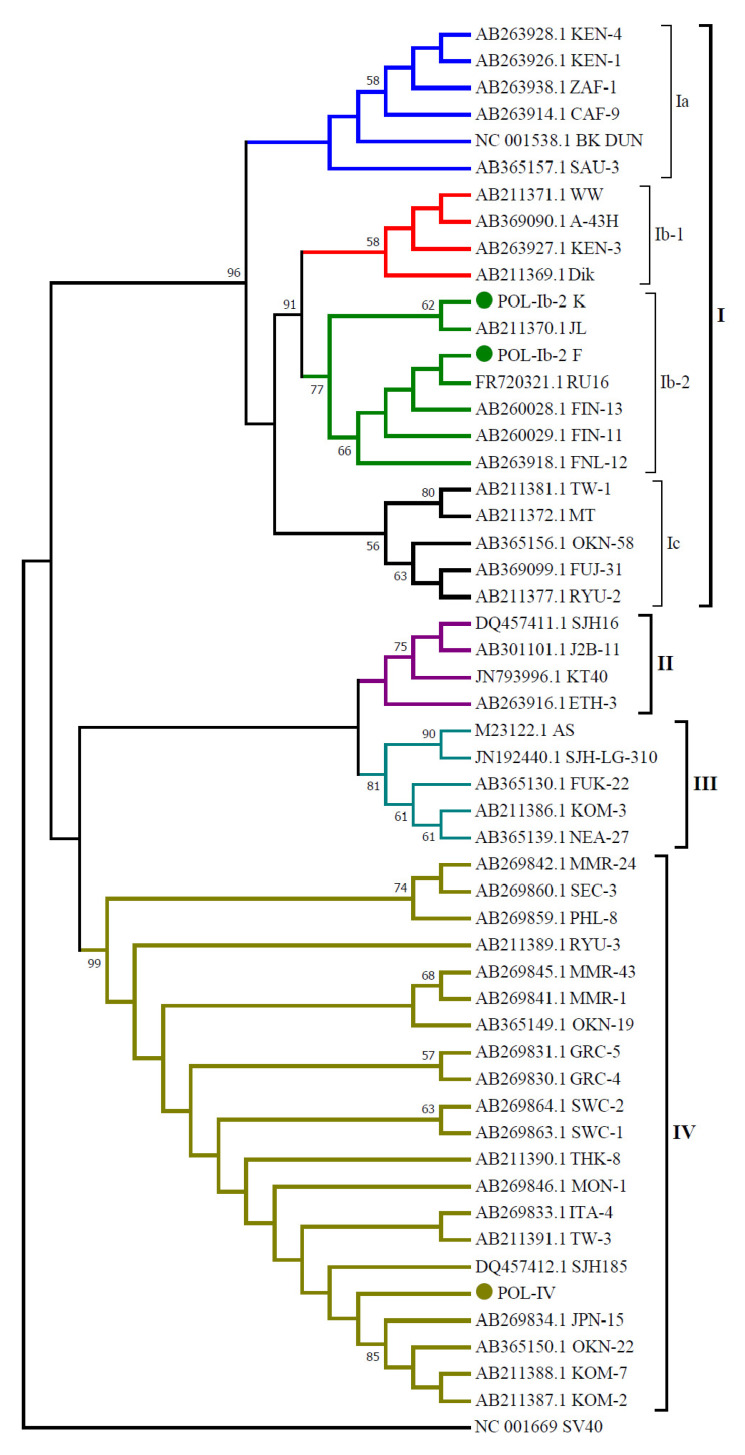
Phylogenetic tree presenting relationships between BKPyV variants obtained in the study and sequences from the GenBank database. Phylogenetic analysis was carried out in MEGA7 software by the neighbour-joining (NJ) algorithm with Kimura’s two-parameter distance method and a bootstrap value of 1000. The analysis was based on a 287 bp fragment of the VP1 coding sequence.

On the basis of phylogenetic analysis, a tree was obtained illustrating the clear division BKPyV into four main genotypes and four subtypes within genotype I. Phylogenetic analysis of variants Ib-2_POL_K and Ib-2_POL_F confirmed that they belong to subtype Ib-2, dominant in Europe (Figure 2). The assignment of the isolates to this subtype is also confirmed by the presence of cytosine at position 1687 and adenine at position 1908, i.e., the nucleotides indicated by Ikegaya as typical of Ib-2 [28]. Our isolates were clearly divided into two groups within subtype Ib-2, which for clarity we designated as Ib-2_POL_K and Ib-2_POL_F. The groups occupied separate branches within the same clade, due to a polymorphism at position 1809 (G1809A and G1809C, respectively) with a bootstrap value of 77% (Figure 4).

Phylogenetic analysis of a fragment of the sequence encoding the protein VP1 did not allow for conclusive identification of the subtype of our isolates classified as genotype IV, designated POL-IV.

Sequence analysis of the variants circulating in Poland, as in the observations of Ikegaya et al. 2006, indicates that nucleotide 1809 may be polymorphic within subtype Ib, and there are variants both with adenine (over 80 sequences in the NCBI database showing 100% similarity to Ib-2_POL_K) and with cytosine (over 30 sequences in the NCBI database showing 100% similarity to Ib-2_POL_F). Sequences with 100% similarity to Ib-2_POL_K, include isolates from Russia (FR720309.1), Spain (JX195567.1), the United States (AB301096.1), and Hungary (AB276160.1), as well as the Dutch isolate JL (AB211370.1). The variant designated in our paper as Ib-2_POL_F has a relatively high frequency in Poland (38.46% of samples with type I were infected with the Ib-2_POL_F variant). Among sequences from the Genbank database, 100% similarity to this variant is found for isolates from Russia (FR720321.1), Belarus (KR075894, unpublished), France (JN793994, unpublished), the United States (AB301100), the United Kingdom (AB263921.1), Finland (AB260028.1), Sweden (AB263935) and Ireland (DQ457406). Comparison of BKPyV variant-POL-IV with the GenBank database reveals that 40 sequences deposited in the database show 100% similarity to POL-IV within the analysed nucleotides. These include isolates from Thailand (MG871519.1), Germany (KF468279.1), France (JN794002.1), Italy (AB269833.1), and Mongolia (AB269846.1) (Table 6).

## 4. Discussion

Serological studies indicate that the BKPyV is widespread in tested populations. Egli et al. [3] showed that specific antibodies against the BKPyV were present in 82% of healthy subjects, but the genetic material of the virus was found in the urine of only 7%. A slightly higher percentage of patients with viruria was reported by Dehcheshmeh et al. [4] in a study of 164 healthy volunteers, in which the presence of BKPyV DNA was confirmed in nearly 13%. Our results indicate a relatively high frequency of viruria, confirmed in 36% of subjects. A similar high percentage of infected subjects was reported by Cobos et al. [36] and by Ducharme-Smith et al. [37], who detected BKPyV in 36% of kidney transplant recipients and 34% of heart transplant recipients. A somewhat lower rate of viruria was obtained by Zhong et al. [38], in a study of outpatients and healthy volunteers; the average frequency of positive amplifications was 27% (14–44%). The authors noted a relationship between age and the incidence of viruria: the rate of BK viruria was relatively low in subjects under the age of 30 years but gradually increased with age in subjects aged ≥30 years. Zhong et al. [38] showed the highest frequency of detection of BKPyV DNA in the group of patients aged 80–89 years at 44%. Dehcheshmeh et al. [4] also noted that the rate of BKV in the 60–79 age group was higher than in the group <20 years. A similar tendency was confirmed in the present study with the highest incidence of BKPyV noted in the group over the age of 60. Age and impaired immunity are predisposing factors for accelerated replication of the virus. Hu et al. [39] showed BKPyV viruria in over 64% of patients infected with human immunodeficiency virus-1, while Karalic et al. [21] detected BKPyV viruria in more than 50% patients infected with HIV and in only 7.5% of healthy donors.

Guidelines from the American Society of Transplantation Infectious Diseases Community of Practice emphasise the importance of evaluating BKPyV DNA in plasma and urine as a valuable prognostic biomarker [40]. In the present study, no genetic material of BKPyV was detected in any of the blood samples. The average viral load in the urine in the population did not exceed 10^5^ copies of BKPyV/mL, which is within recommended limits, as the threshold value of urine viral load is 1 × 10^7^ copies/mL [41]. In healthy individuals, asymptomatic reactivation with viruria may occur, which is of no clinical significance and is not associated with any urological problems what is also confirmed by our results.

Bioinformatic analysis of the sequences enabled the identification of genotypes and subtypes of the virus. Sharma et al. [42], citing Chen et al. [43], suggest that positions 1698, 1809, and 1923 are significant for identification of subtype Ib, which is also confirmed by our own results. The researchers also indicate the potential importance of the G1702C polymorphism, which may be associated with tropism of the virus for brain tissue, but this polymorphism was not found in any of the Polish variants.

In the present study there was a high percentage of isolates representing genotype IV. Nishimoto et al. [26], using the TW-3 sequence (AB-211391) as a reference, identified polymorphic nucleotides allowing genotype IV to be divided into subtypes. The amplified region contained three of these nucleotides. The sequences obtained with genotype IV had adenine at nucleotide position 1732, which is characteristic of all isolates belonging to genotype IV. The presence of guanine at position 1741 indicates that the isolate belongs to subtype IVb or IVc, while the presence of thymine at position 1807 rules out the occurrence of subtype IVb-2. Unfortunately, the analysis of polymorphism in the typing region (1630–1956 bp) did not allow for precise classification of isolates with genotype IV to a specific subtype. Observations by Nishimoto et al. [26] indicate that the dominant subtype in Europe is IVc-2, and thus it is very likely that the variants obtained in our study can also be included in this subtype. In our study there were no representatives of genotypes II or III, which are relatively rare on a global scale [44].

The results of our analysis of molecular variation in BKPyV indicate that genotype I is dominant in the Polish population. A similar tendency is noted in most European countries. Studies on the molecular epidemiology of individual genotypes and subtypes of BKPyV have been conducted in England, Ireland and Germany, where the frequency of genotype I was 88–90%, while genotype IV was noted in only 3% of subjects in Ireland [45] and England [46]. Research on molecular variation in the BKPyV in the German population found that genotype I was present in over 90% of samples. Interestingly, in contrast with the majority of European countries, where subtype Ib is the most common, the dominant subtype in Germany was Ic [47]. A higher proportion of genotype IV is observed in Finland. Ikegaya et al. [28] confirmed the presence of genotype IV in over 29% of samples. A similar tendency was observed in Russia, where genotype IV was detected in 24% of subjects [48]. Research conducted in Serbia demonstrated the dominance of genotype I in both HIV-infected and healthy individuals, while the percentage of genotype IV was 19% in HIV-positive subjects and over 33% in the group of healthy volunteers [21].

Varied ratios of genotype I to IV are also indicated by results presented by Zheng et al. [24], who did not detect genotype IV in Great Britain or Spain, whereas in Finland, Greece and the Czech Republic the number of isolates with genotypes I and IV was similar, and in Hungary genotype IV was clearly dominant. However, the sample size was too small to allow for detailed conclusions regarding individual countries. The researchers determined the average frequency of genotype IV in Europe to be 39% [24].

There are two hypotheses regarding the genesis of genotype IV of the BKPyV, which is the dominant variant in eastern Asia [25]. According to one theory, the virus migrated with the population from Africa to Asia, while the other suggests that the pathogen arose after the colonization of Asia, which was its original source. The relatively low incidence of genotype IV in African countries suggests an Asian origin and colonization of other continents together with the migration of the hosts [26]. The view that subtype IV originated in East Asia was supported by a phylogenetic analysis addressed to complete subtype IV genomes worldwide [26]. In the phylogenetic tree of 52 complete BKPyV genome sequences [24], subtype IV forms, with subtypes I and II, a deep ancestral clade. As population contact and interbreeding occurred rather frequently between ancestral modern humans and archaic hominins, subtype IV could have been transmitted from an archaic hominin population in Asia to an ancestral population that generated modern Asians [26,27].

The hypothesis of the transfer of genotype IV is also supported by its more frequent occurrence in Eastern Europe than in the western part of the continent. Research in the Finnish population showed unequal proportions of genotypes I and IV in different parts of the country. Research on the molecular epidemiology of BKPyV also shows a high percentage of isolates representing genotype IV in Greece and Hungary [26]. Chen et al. [25] showed that genotype IV was present in most diagnosed samples from north-western China (65%), south-western China (88%), Vietnam (53%), and Mongolia (100%), while the presence of BKPyV IV in Japan was low (8%).

Our results indicate a relatively high frequency of BKPyV genotype IV in the Polish population, as in Finland, Russia, and Hungary. In Europe, the most commonly detected subtype within type IV is IVc-2. Contemporary international and transcontinental population flow is conducive to the spread of the pathogen, and for this reason type IV is also diagnosed in the United States [29]. The appearance of IV in America was most likely caused by the migration of human hosts of the pathogenfrom Asia and Europe [26].

The synonymous mutation G1809C detected in our study had already been observed by researchers such as Ikegaya et al. [28], and Zheng et al. [24], but in a relatively small number of isolates. In our test population, the frequency of variant G1809C, designated in the paper as Ib-2_POL_F, reaches 24.59%, which justifies its distinction as a separate subclade within subtype Ib-2. The differences between variants within subtype Ib-2 are also confirmed by the dendrogram, with a bootstrap value of 77 between subclades G1809C and G1809A.

The frequency of variant Ib-2_POL_K was higher in older age groups, while variant Ib-2_POL_F did not exhibit such a dependency, with similar frequency in all age groups. Both Ib-2_POL_F and Ib-2_POL_K were more common in men than in women. Neither of the Ib-2 variants caused clinical symptoms, and their carriers had not noticed their presence.

Nonsynonymous changes in the nucleotide sequence, as in the case of Ib-2_POL_K, entail changes in the amino acid sequence, which can translate to changes in the structure and activity of the protein. In the case of the JC polyomavirus, a close relative of BKPyV, nonsynonymous mutations in the BC and HI loops of the VP1 protein may be linked to the occurrence of progressive multifocal leukoencephalopathy (PML) [49]. Research by Karalic et al. [21] indicates that the E82D polymorphism (occurring in the variant designated in our paper as Ib-2_POL_K) was the mutation most commonly noted among BKPyV isolates in patients additionally infected with HIV. Synonymous mutations, where the amino acid sequence remains unchanged (variant Ib-2_POL_F), can also affect the rate of expression and thus the replication of the pathogen and generation of an immune response. In BKPyV infections, higher molecular variation has been observed in variants isolated from patients with BKPyVAN than from healthy donors or recipients in which the graft was not destroyed [34]. Moreover, it has been reported that, due to great selective pressure, polyomaviruses show a tendency to accumulate mutations in VP1 capsid epitopes in order to evade neutralizing antibodies [21,50]. Given the above, not only detection of the virus in populations is justified, but also molecular analysis, as both synonymous and nonsynonymous mutations can be associated with an unfavourable course of BKPyVAN, if a kidney is transplanted from an infected donor to a BKPyV-free recipient, and with the course of the disease in patients with impaired immunity.

## 5. Conclusions

The paper presents the first results of analysis of the epidemiology of the BKPyV in the Polish population, indicating dominance of type I as well as a high percentage of type IV. Two variants were detected within subtype Ib-2, differing in a single nucleotide substitution (A1809C). The significant molecular variation in the pathogen, the large number of type IV isolates, and the scarcity of data on the epidemiology of BKPyV in Poland suggest that further research should be conducted to provide a more detailed characterization of the pathogen and to investigate its relationships with isolates from other countries.

## Figures and Tables

**Table 1:** Primer sequences used for PCR, product lengths obtained after amplification, and references to the studies in which the primers were first used.

Primer Name		Primer Sequence	Product Length	References
BKPyV	327-1PST	CAAGTGCCAAAACTACTAAT	327 bp	[28,33]
	327-2HIN	GCATGAAGGTTAAGCATGC		
β-globin	PC03_F	ACACAACTGTGTTCACTAGC	110 bp	[32]
	PC04_R	CAACTTCATCCACGTTCACC		

**Table 2:** Reaction mix composition and temperature profiles for each primer pair.

Reaction Mix Composition	BKPyV		β-Globin	
Water	to 25 µL		to 25 µL	
Buffer 10×	1× concentrated		1× concentrated	
GC enhancer	0.04 in final concentration		0.04 in final concentration	
Mg^2+^	2.5 mM		2.5 mM	
dNTP	0.8 mM		0.8 mM	
Forward primer	0.8 µM		0.8 µM	
Reverse primer	0.8 µM		0.8 µM	
Polymerase	1U		1U	
Temperature profile	Temperature	Time	Temperature	Time
Initial denaturation	95 °C	10 min	95 °C	10 min
Denaturation	95 °C	45 s	95 °C	45 s
Annealing	54 °C	45 s	56 °C	45 s
Elongation	72 °C	45 s	72 °C	45 s
Final elongation	72 °C	10 min	72 °C	10 min

**Table 3:** Frequency of occurrence of BKPyV in the study group by age and sex.

	Without BK		Confirmed Presence of BKPyV		Total	
Sex	Number	Age	Number	Age	Number	Age
Women	36	56 (29–72)	22	53 (27–67)	58	55 (27–75)
Men	71	47 (21–73)	39	53 (25–75)	110	49 (21–75)
Total	107		61		168	

**Table 4:** Location of polymorphic nucleotides in relation to the sequence of the reference strain BK DUN (NC_001538.1_BK_DUN); red colour—nucleotides distinguishing types I and IV, bold—nucleotides specific for subtype Ib, orange background—nucleotide distinguishing variants Ib-2_POL_K and Ib-2_POL_F.

	NC_001538.1_BK_DUN	Ib-2_POL_K	Ib-2_POL_F	POL-IV
1687	G	**C**	**C**	
1698	T	**A**	**A**	A
1704	G			A
1716	C			T
1722	C			T
1744	G			A
1746	A			T
1747	A			G
1760	T			A
1769	A			G
1770	G			A
1775	G			C
1784	A			C
1787	A			C
1792	A			G
1793	G			A
1809	G	**A**	**C**	C
1848	C			A
1851	C			A
1854	C			T
1860	A			G
1869	C			T
1890	G			A
1905	A			G
1908	T	**A**	**A**	
1912	C			A
1923	T	**C**	**C**	

**Table 5:** Viral load and distribution of subtypes of the virus depending on age and sex.

Sex	Age	Number	Average Number of Copies	Type		
				Ib-2_POL_K	Ib-2_POL_F	POL-IV
M	21–30	2	7.81 × 10^3^	0	1	1
	31–40	6	1.55 × 10^6^	2	3	1
	41–50	8	2.27 × 10^4^	4	2	2
	51–60	9	2.82 × 10^3^	5	2	2
	61–70	9	5.63 × 10^4^	4	1	4
	71+	5	6.65 × 10^3^	1	2	2
Total		39		16 (41.02%)	11 (28.21%)	12 (30.77%)
F	21–30	2	1.18 × 10^4^	1	0	1
	31–40	1	8.00 × 10^2^	1	0	0
	41–50	7	1.50 × 10^4^	2	1	4
	51–60	3	6.56 × 10^4^	1	1	1
	61–70	9	3.10 × 10^5^	3	2	4
Total		22		8 (36.36%)	4 (18.18%)	10 (45.45%)
M and F	21–30	4	9.78 × 10^3^	1	1	2
	31–40	7	1.33 × 10^6^	3	3	1
	41–50	15	1.91 × 10^4^	6	3	6
	51–60	12	1.85 × 10^4^	6	3	3
	61–70	18	1.83 × 10^5^	7	3	8
	71+	5	6.65 × 10^3^	1	2	2
Total		61		24 (39.34%)	15 (24.59%)	22 (36.07%)

**Table 6:** Degree of similarity of the fragment of the VP1 coding sequence of BKPyV between Polish isolates and selected sequences from the GenBank database.

Sequence	Ib-2_POL_K	Ib-2_POL_F	POL-IV	Type
AB276214.1_CAF-9	97.90%	97.90%	91.60%	Ia
AB263938.1_ZAF-1	97.90%	97.90%	91.60%	
AB365157.1_SAU-3	96.80%	96.80%	91.20%	
NC_001538.1_DUN	98.20%	98.20%	91.60%	
AB211369.1_Dik	99.30%	98.90%	91.60%	Ib-1
AB211371.1_WW	98.90%	98.60%	91.20%	
AB263927.1_KEN-3	99.30%	98.90%	91.60%	
AB369090.1_A-43H	99.30%	98.90%	91.60%	
AB211370.1_JL	100.00%	99.60%	90.90%	Ib-2
AB276160.1_HUN-2	100.00%	99.60%	90.90%	
AB276157.1_CZE-4	100.00%	99.60%	90.90%	
AB260029.1_FIN-11	99.60%	100.00%	91.20%	
AB260028.1_FIN-13	99.60%	100.00%	91.20%	
FR720321.1_RU16	99.60%	100.00%	91.20%	
AB211372.1_MT	97.90%	97.90%	91.90%	Ic
AB211381.1_TW-1	97.50%	97.50%	91.60%	
AB211377.1_RYU-2	97.20%	97.20%	91.90%	
AB369099.1_FUJ-31	97.90%	97.90%	92.60%	
JN793996.1_KT40	93.70%	94.00%	94.70%	II
AB263916.1_ETH-3	94.40%	94.70%	94.00%	
DQ457411.1_SJH16	93.70%	94.00%	94.70%	
AB301101.1_J2B-11	93.70%	94.00%	94.70%	
AB365139.1_NEA-27	91.60%	91.90%	93.30%	III
M23122.1_AS	91.90%	92.30%	92.60%	
AB211386.1_KOM-3	91.20%	91.60%	93.00%	
JN192440.1_SJH-LG-310	92.30%	92.60%	92.30%	
DQ457412.1__SJH185	90.90%	91.20%	100.00%	IV
AB269859.1_PHL-8	90.90%	91.20%	98.90%	
AB365150.1_OKN-22	90.20%	90.50%	99.30%	
AB269834.1_JPN-15	89.80%	90.20%	98.90%	
AB269863.1_SWC-1	91.20%	91.60%	99.60%	
AB269846.1_MON-1	90.90%	91.20%	100.00%	
AB269830.1_GRC-4	90.50%	90.90%	99.60%	
AB269833.1_ITA-4	90.90%	91.20%	100.00%	
AB269864.1_SWC-2	91.20%	91.60%	99.60%	
AB269831.1_GRC-5	90.50%	90.90%	99.60%	

## Data Availability

Obtained sequences are deposed in NCBI under accession numbers: OM179842 (Ib-2_POL_K), OM179843 (Ib-2_POL_F), OM179844 (POL-IV).

## Supplementary Materials

The following supporting information can be downloaded at: https://www.mdpi.com/article/10.3390/v14020209/s1, Table S1: Viral load and distribution of subtypes of the virus depending on age. Table S2: Viral load and distribution of subtypes of the virus depending on sex.
