# Supplementary material for: Molecular Epidemiology and Variation of the BK Polyomavirus in the Population of Central and Eastern Europe Based on the Example of Poland
Source: Viruses. 2022 Jan 21;14(2):209. doi: 10.3390/v14020209 (PMC8878621; doi:10.3390/v14020209)
Supplement: Supplementary file 1 [file viruses-14-00209-s001.zip › viruses-1554928-supplementary.pdf]

Table S1. Viral load and distribution of subtypes of the virus depending on age.

Age	Number	Average number of copies	Type		
			Ib-2_POL_K	Ib-2_POL_F	POL-IV
21-30	4	9.78E+03	1	1	2
31-40	7	1.33E+06	3	3	1
41-50	15	1.91E+04	6	3	6
51-60	12	1.85E+04	6	3	3
61-70	18	1.83E+05	7	3	8
71+	5	6.65E+03	1	2	2
Total	61	2.16E+05	24	15	22

Table S2. Viral load and distribution of subtypes of the virus depending on sex.

Sex	Number	Average number of copies	Type		
			Ib-2_POL_K	Ib-2_POL_F	POL-IV
M	39	2.58E+05	16	11	12
F	22	1.42E+05	8	4	10
Total	61	2.16E+05	24	15	22
